# Supplementary material for: The Biological Behaviors of Neural Stem Cell Affected by Microenvironment from Host Organotypic Brain Slices under Different Conditions
Source: Int J Mol Sci. 2023 Feb 20;24(4):4182. doi: 10.3390/ijms24044182 (PMC9964775; doi:10.3390/ijms24044182)
Supplement: Supplementary file 1 [file ijms-24-04182-s001.zip › ijms-2182726-supplementary.pdf]

Supplementary materials

Material and methods

1. Assessment of survival and proliferation of cultured NSCs

NSCs and NSCs-EGFP were cultured in growth medium and cell counting kit-8 (CCK-8, Dojindo, JPN) was applied to analyze cell viability and proliferation. Cell viability was observed every day for 5 days by using an UV-1750 spectrometer reader (Shimadzu, JPN) at 450 nm. At the same time, the diameters of 30 neurospheres obtained from three individual wells were measured using IPP software to reveal the survival and the proliferation of NSCs. In addition, proliferation of NSCs was also assessed by Ki-67 expression assay. Neurospheres were trypsinized and mechanically triturated into single cells on 5th day and fixed with 4% PFA at room temperature for 30 min.

2. Propidium iodide uptake and quantification of cell death

Cell damage was quantified by the cellular uptake of propidium iodide (PI, Sigma, St. Louis, MO, USA), a fluorescent dye that intercalates into the DNA of necrotic cells. Before treatments, 5 μg/mL PI was added into the culture medium for 1 h and kept at the same concentration throughout the experiment. The PI uptake was recorded at different time points of treatments. All cells were killed after fixation by 4% PFA at 4°C for 30 min, and the maximum PI uptake served as positive controls for each slice. The PI uptake was quantified densitometrically using IPP software.

3. Lactate dehydrogenase ELISA Assay

Medium from three individual wells were collected for lactate dehydrogenase (LDH) assay via ELISA (Ray Biotech, Atlanta, GA, USA). Following the instruction, a volume of 50 μL medium was added to appropriate well and then incubated with 100 μL HRP-conjugated reagents for 60 min at 37°C. After thoroughly wash, chromogen solutions were added and incubate at 37°C. The reaction was stopped 15 min later and the result was read at 450 nm in an EL808 Ultra Microtiter plate Reader (BIO-TEK instruments, Winooski, VT, US).

Results

S1. The culture of organotypic brain slice.

The survival and growth of brain slices were observed daily under phase contrast microscope (Figure S1A). On the second day of culture, microglia (red arrows) with poor refractive ability could be seen on the brain slices. With the prolongation of culture time, the number of microglia cells increased until 10 DIV. At the same period, a large number of cells migrated outward (yellow arrows) and resulted in the thickness of brain slices gradually reduced. After cultured for 14 days, the original structure of brain was well kept and cells were still viable (Figure S1B). Different types of neural cells were detectable as showed by NF200 and GFAP positive neurons and astrocytes (Figure S1B).

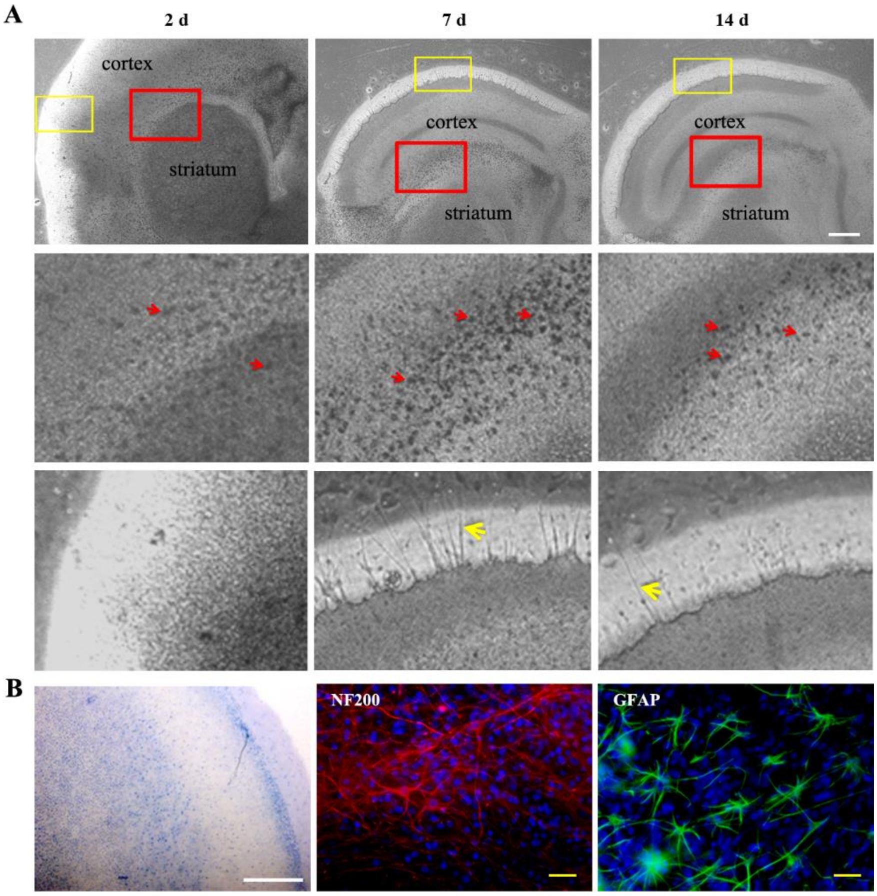

Figure S1. The organotypic brain slice culture. A: The growth and structure alteration of organotypic brain slice at different time. Microglia showed by red arrows and migrating cells showed by yellow arrows. **B:** Stucture and viablity of brain slices, stained with trypan blue, anti-NF

200 and anti-GFAP, respectively. Scale bars= 500 μm (white) and 20 μm (yellow).

S2. The characteristics of NSCs-EGFP

Two days after transduction, NSCs were observed under inverted fluorescent microscope (Figure S2A). Our results demonstrated that 91.01% ± 3.227 of NSCs expressed EGFP which indicated a successful transduction (Figure S2B). The characteristics of NSCs-EGFP in forming neurospheres and undergo multiple differentiation were then determined. Different sizes of spherical cell clusters emerged after 2 days in growth medium, and the immunocytochemical staining confirmed that most of EGFP labeled cells were nestin- or ki-67-positive neural progenitors (Figure S2C) and those cells could differentiated into $GFAP^+$ astrocytes and β-tubulin III^+ neurons after seeding into differentiation medium (Figure S2D). This indicated that the EGFP- labeled cells maintained NSCs characteristics. CCK-8 assay showed that NSCs-EGFP survived well and no significant differences was found when compare with normal control group (NC) (Figure S2E).

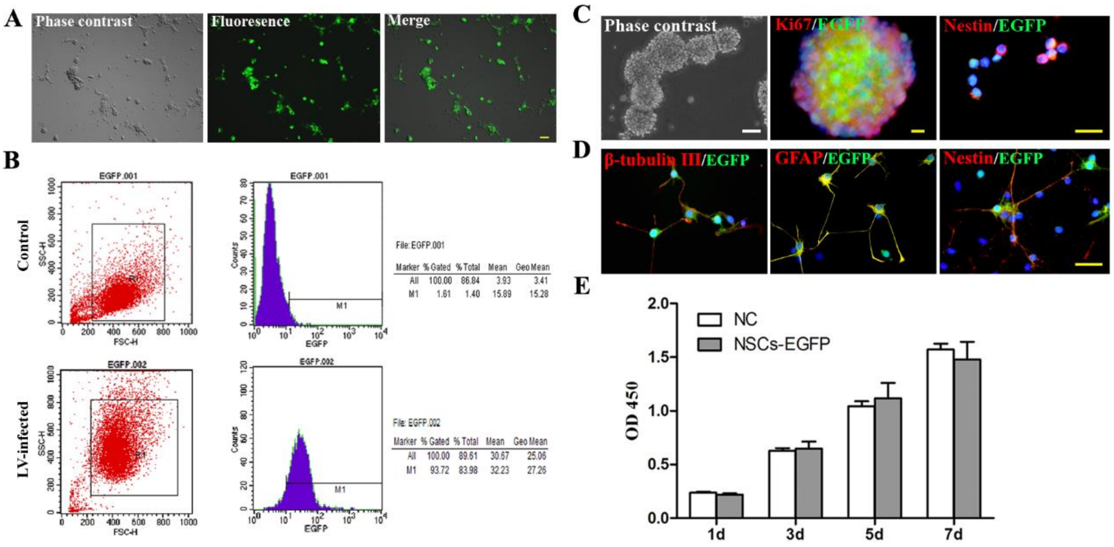

Figure S2. The characteristics of NSCs-EGFP. A: NSCs were observed under inverted fluorescent microscope after transduction for 2 days. Scale bars= 50 μm. **B**: The percentage of EGFP positive cells was more than 90%. **C and D**: The NSCs ability was well maintained after transduction. NSCs-EGFP formed neurospheres. Most of them were nestin- or Ki-67-positive neural progenitors. Some of them differentiated into GFAP-positive astrocytes and β-tubulin III-positive neurons in differentiation medium. Scale bars= 50 μm. **E:** No significant difference was detected in

regarding cell viability detected by CCK-8 kit. The values are mean ± SE.

S3. The morphological alteration of brain slice after OGD damaged

Whole brain slices cultured for 14 days were exposed to OGD for 0.5 h, 1h and 2 h, respectively. Differ with the slices before OGD (OGD 0 h), the border of different brain regions was hard to distinguish, while the number of dead cells, which stained with PI significantly increased after cultured in OGD condition for 1 h or 2 h (Figure S3A-B). LDH releasing was dramatically up regulated (Figure S3C, *P*<0.001). Astrocytes in slices were activated and the morphology were clearly changed. The processes of astrocytes became bolder and bend, then further twining each other (Figure S3D, yellow arrows). Karyopyknosis were observed (Figure S3D, white arrows) and cell apoptosis (Figure S3E), showed by caspase3 level, and increased with OGD.

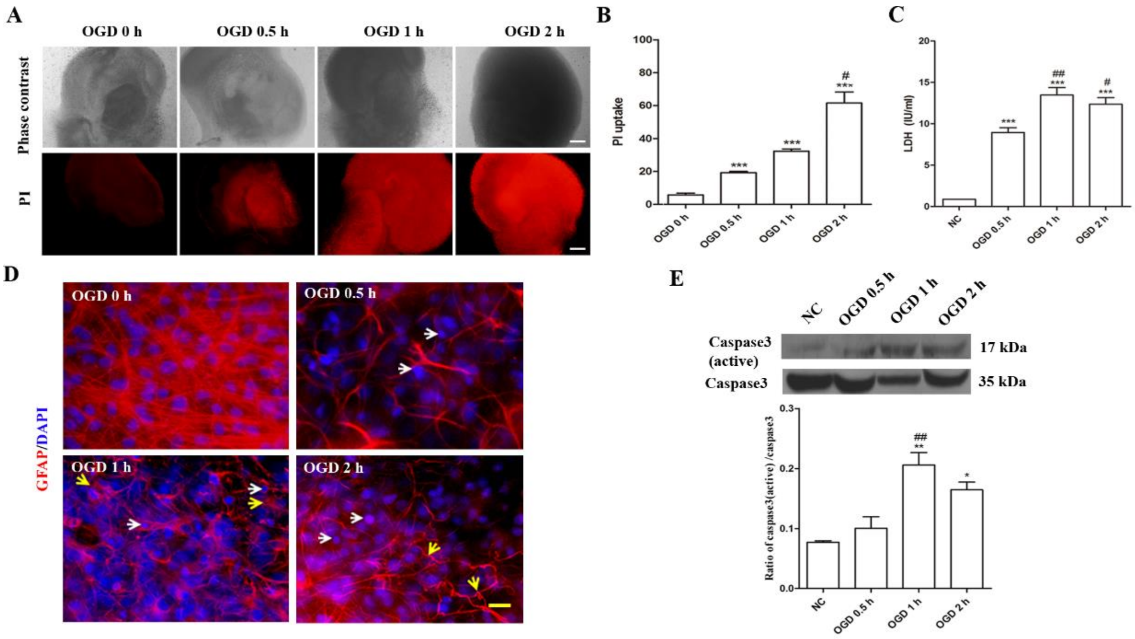

Figure S3. The morphological alteration of brain slices in OGD model. A: The survival status of brain slices after OGD treated for 0.5 h, 1 h, and 2 h. Scalar bar= 500 µm. **B**: Statistic analysis of PI staining cells. The values are mean ± SE. Compared with OGD 0 h, ****P*< 0.001; compared with OGD 0.5 h, #*P*< 0.05. **C**: LDH releasing after OGD treatment. The values are mean ± SE. Compared with OGD 0 h, ****P*< 0.001; compared with OGD 0.5 h, #*P*< 0.05, ##*P*< 0.01. **D**: The morphological changes of GFAP-positive astrocytes after OGD treatment. White arrow: karyopyknosis; yellow arrows: twining processes. Scalar bar= 20 µm. **E**: The level of active-caspase3. The values are mean ± SE. Compared with OGD 0 h, **P*< 0.05, ***P*< 0.01; compared

with OGD 0.5 h, ##P< 0.01.

S4. Astrocytic response of organotypic brain slices after mechanical trauma

Mechanical traumas were established by stabbing the brain slices with the fine end of yellow plastic tips after 14 days culture (Figure S4A). One day after, dramatically raised level of LDH (Figure S4B, P <0.001) and clearly tissue loss, which looked like a 'valley' (marked with red line, Figure S4C) were detected and that conformed the traumatic injury. Along with the culture, brain slices became thinner due to the active cell migration. In addition, strong astrocytes activation was observed around the border of valley-like tissue loss. The morphology of GFAP positive cells that around the injury were significantly different with that in the undamaged slices and all the process extended trimly toward the center of trauma (Figure S4C).

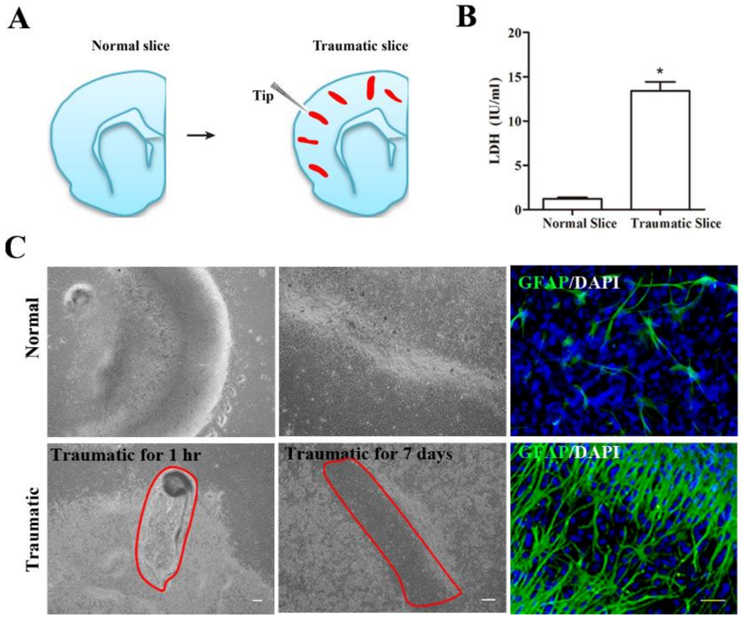

Figure S4. The astrocytic response of organotypic brain slice after mechanical trauma. A: The schematic diagram of mechanical traumas in cerebral cortex (blue region). Red regions represented the mechanical traumas created by fine end of tip. **B**: LDH level was significantly increased after traumatic injury. The values are mean ± SE, * P< 0.001. **C**: Morphological alteration of brain tissue (marked by red lines) and astrocytic response after mechanical traumas. Scale bars with white color, 200 μm. Scale bars with yellow color, 50 μm.
